# A Meta-Analysis of the Effects of Mental Contrasting With Implementation Intentions on Goal Attainment

**DOI:** 10.3389/fpsyg.2021.565202

**Published:** 2021-05-12

**Authors:** Guoxia Wang, Yi Wang, Xiaosong Gai

**Affiliations:** School of Psychology, Northeast Normal University, Changchun, China

**Keywords:** mental contrasting, implementation intentions, goal attainment, self-regulation, meta-analysis

## Abstract

Mental contrasting with implementation intentions (MCII) is a self-regulation strategy that enhances goal attainment. This meta-analysis evaluated the efficacy of MCII for goal attainment and explored potential moderators. A total of 21 empirical studies with 24 independent effect sizes (15,907 participants) were included in the analysis. Results showed that MCII to be effective for goal attainment with a small to medium effect size (g = 0.336). The effect was mainly moderated by intervention style. Specifically, studies with interventions based on interactions between participants and experimenters (g = 0.465) had stronger effects than studies with interventions based on interactions between participants and documents (g = 0.277). The results revealed that MCII is a brief and effective strategy for goal attainment with a small to moderate effect; however, because of some publication bias, the actual effect sizes may be smaller. Due to small number of studies in this meta-analysis, additional studies are needed to determine the role of moderator variables.

## Introduction

People face many obstacles in pursuit of their goals. Self-regulation interventions, using strategies such as mental contrasting and implementation intentions, have become popular in promoting successful goal attainment.

### Mental Contrasting and Its Effects on Goal Attainment

Several studies by Oettingen and colleagues found that thinking only about positive future outcomes decreases goal-relevant efforts, as well as the likelihood of goal achievement (e.g., Oettingen and Mayer, 2002; Kappes H. B. et al., 2012; Oettingen et al., 2016). After imagining a positive future, thinking about obstacles that impede the realization of wishes (i.e., mental contrasting) can transform people's positive fantasies into binding goals, urging them to overcome obstacles in reality to attain their goals (Oettingen, 2000). Mentally contrasting a desired future with impeding reality can activate expectations of success: when expectations of success are high, people fully commit and pursue their goals; when expectations of success are low, people postpone or abandon the fulfillment of their wishes (Oettingen, 2000, 2012). Effects of mental contrasting on goal pursuit are mediated by three non-conscious processes: cognition, energization, and response to feedback. In terms of cognitive mechanisms, mental contrasting can strengthen non-conscious mental associations between the future and reality (Kappes and Oettingen, 2014), help individuals identify relevant obstacles in their current reality (Kappes et al., 2013), and increase the implicit mental link between real obstacles and the instrumental behaviors needed to overcome them (Kappes H. B. et al., 2012). Oettingen et al. (2009) found that energization (measured both by an implicit indicator, namely, systolic blood pressure, and by self-report) mediated the effects of mental contrasting on effort and performance. Finally, Kappes et al. (2012a) found that mental contrasting facilitated effective responses to negative feedback, in line with expectations of success.

A series of intervention studies showed that mental contrasting could improve participants' academic performance, dietary behavior, and physical activities (reviewed by Oettingen, 2012). A meta-analysis by Cross and Sheffield (2019) found a main effect of a mental contrasting intervention on adults' physical health outcomes, with adjusted Hedges' *g* = 0.28, 95% CI (0.13, 0.43) at up to 4 weeks, and an increased effect [*g* = 0.38, 95% CI (0.20, 0.55)] at up to 3 months.

### Implementation Intention and Its Effects on Goal Attainment

Goal intention alone does not guarantee the success of actions; goal setting should be followed by planning. Implementation intention is an if–then plan that specifies when, where, and how the behavior will lead to the achievement of a goal (e.g., if situation *y* arises, then I will perform goal-directed behavior *z* to achieve goal *x*) (Gollwitzer, 1999). Implementation intentions promote accessibility of situation cues (Sheeran and Webb, 2004) and strengthen the links between the anticipated situation and the expected behavior so that, upon encountering the relevant cues, goal-relevant behavior would appear automatically (Webb and Sheeran, 2007). Thus, implementation intentions narrow the gap between goal and action and optimize goal pursuit.

Several meta-analytic studies evaluated the effect size of implementation intentions. For example, Gollwitzer and Sheeran (2006) found that implementation intentions had a positive effect on improving goal attainment in terms of achievements, relationships, and health, with a medium to large effect size [Cohen' *d* = 0.65 (95% CI = 0.6, 0.7)]. Presseau et al. (2017) found that action and/or coping planning (implementation intention) interventions had a small to medium effect on objectively assessed health behavior (Cohen's *d* ranged from 0.14 to 0.37) and a medium effect on self-reported health behavior (Cohen's *d* ranged from 0.19 to 0.77). Adriaanse et al. (2011) found implementation intention interventions to have a moderate effect size for promoting the inclusion of healthy food items in one's diet (Cohen's *d* = 0.51) and smaller effects for diminishing unhealthy eating patterns (Cohen's *d* = 0.29). Belanger-Gravel, Godin and Amireault's (2013) meta-analysis of implementation intention interventions on physical activity found small to medium effect size [Hedge's *g* = 0.31 (95% CI = 0.11, 0.51) at postintervention; Hedge's *g* = 0.24 (95% CI = 0.13, 0.35) at follow-up]. In total, implementation intentions have a small to large effect on behavior change.

### The Effect of Mental Contrasting With Implementation Intentions on Goal Attainment

Mental contrasting is a goal-setting strategy that can transform positive fantasy into binding goal commitment, followed by goal striving; implementation intention is a goal implementation strategy that supplements goal intention and drives action. A combination of mental contrasting and implementation intentions may bolster goal pursuit. Mental contrasting can not only lead to binding goals and provide goal commitment as a premise for implementation intentions (Sheeran et al., 2005) but also help people to identify obstacles in reality and to create non-conscious links between obstacles and instrumental behavior while engendering a readiness for the establishment of implementation intentions (Kappes et al., 2012b, 2013). Even if people form strong goal commitment by mental contrasting, they are not always able to successfully turn it into goal-directed behavior. For example, people may forget to act or encounter difficult obstacles, such as impulses, strong emotions, or ingrained habits, or they may be unaware of potential action cues (Oettingen, 2012). Implementation intentions are additional strategies for overcoming such challenges, as they strengthen the association between obstacles and goal-relevant instrumental behaviors to overcome obstacles, which is created through mental contrasting (Gollwitzer, 1999; Kappes et al., 2012b). Both laboratory experiments and field studies have shown that combining mental contrasting with implementation intentions is more effective in goal pursuit than either mental contrasting or implementation intentions (Adriaanse et al., 2010; Kirk et al., 2013). However, unlike most implementation intentions studies, where researchers provide the effective content to be inserted in if–then plans, mental contrasting and mental contrasting with implementation intentions (MCII) studies allow participants to autonomously define their desired future, identify obstacles existing in reality, and formulate an “if obstacle, then I will behavior” plan. Although the autonomous use of MCII decisively increases the scale of application in everyday life, participants may generate disappointing outcomes, formidable obstacles, or ineffective plans for overcoming obstacles (Kizilcec and Cohen, 2017), which might reduce the effect of the mental contrasting and MCII interventions. Still, a meta-analysis of health-related behavior by Cross and Sheffield (2019) found that a combination of mental contrasting and implementation intentions showed a small-to-medium effect, *g* = 0.28, 95% CI (0.14, 0.42), similar to the effect size of a mental contrasting only intervention. There is, therefore, a need for a meta-analysis study of MCII interventions on all types of goals, not only those related to health, to evaluate its effect size.

MCII intervention studies increased in recent years, and most studies have shown that it can improve goal attainment (e.g., Stadler et al., 2009, 2010). A few studies have found MCII to be effective under certain circumstances. For example, Kizilcec and Cohen (2017) found that MCII could increase individual goal attainment (massive open online courses, abbreviated by MOOCs, completion rates) in individualist cultures but not collectivist cultures. Sailer et al. (2015) found that MCII improved patient' exercise in autonomy-focused settings, but not in a highly structured setting, compared to control condition. Wittleder et al. (2019) found that MCII decreased the number of drinking days for people with hazardous drinking behavior, but not for people with non-hazardous drinking behavior, compared to the control group. Thus, a meta-analysis is needed to assess the effect of MCII on goal achievement beyond the scope of health behavior and explore potential moderators.

We expect that the MCII intervention effect on goal attainment might be small to medium, although mental contrasting with implementation intentions is more effective on goal pursuit than either mental contrasting or implementation intention (Adriaanse et al., 2010; Kirk et al., 2013). First, in contrast to meta-analyses that included laboratory, related, and intervention studies that found medium to large effect size (Gollwitzer and Sheeran, 2006), meta-analytical studies have found that mental contrasting interventions alone (Cross and Sheffield, 2019), or implementation intentions interventions alone (e.g., Adriaanse et al., 2011; Presseau et al., 2017), had small to medium effect sizes on goal attainment. Second, the effects of both mental contrasting and implementation intentions are moderated by other variables (e.g., Churchill and Jessop, 2010; Oettingen and Cachia, 2016). The combination of these two strategies will be moderated by more variables, which may decrease the effect size. Lastly, a meta-analysis by Cross and Sheffield (2019) found that the effect size of MCII interventions was similar to that of a mental-contrasting-only intervention in terms of health-related behavior.

### Potential Moderating Variables on the Effect of MCII on Goal Attainment

In the current study, we examined six potential moderating variables: publication status, sample age, goal domain, type of intervention, dependent measures, and goal success expectation.

#### Publication Status

Studies with significant results could be easier to publish, so published articles might overestimate the effect of MCII. To avoid such publication bias, we included unpublished master's theses and doctoral dissertations in the analysis and examined the difference in effect size between published and unpublished studies.

#### Sample Ages

Most MCII intervention studies have been conducted among adults, although few have sampled children (Duckworth et al., 2011, 2013). Participants in intervention studies with adults included college students, adults with physical or psychological illnesses, and adults from the general population (Christiansen et al., 2010; Oettingen et al., 2015; Gollwitzer et al., 2018).

#### Goal Domain

MCII interventions have been used in four different goal domains, namely, the academic domain, such as preparing for high-stakes exams (Duckworth et al., 2011) and completing online courses (Kizilcec and Cohen, 2017); the health domain, such as physical activity (Christiansen et al., 2010), healthy diet (Adriaanse et al., 2010), and stress management (Gollwitzer et al., 2018); the relationship domain, such as romantic relationships (Houssais et al., 2013) and conformity (Scheurnschloß, 2017); and personal goal domain, including goals that were not limited to a specific goal domain (Wang and Gai, 2016) and personal time management (Oettingen et al., 2015). The current study explored the moderating effect of goal domains.

#### Type of Intervention

Two types of intervention procedures were identified. The first is face-to-face interventions with experimenters, or “experimenter intervention,” usually conducted in a laboratory, hospital, or rehabilitation center, and comprising a meeting between one experimenter and one participant or between one experimenter and a small group of participants (Stadler et al., 2009; Oettingen et al., 2015). The second type of interventions is a “document intervention,” which includes completing the intervention package online, at home, or in the classroom (Duckworth et al., 2011; Kizilcec and Cohen, 2017). As face-to-face interactions have been found to have a better effect on behavior change than documents interventions (Elder et al., 2005), we hypothesized that experimenter interventions would have a larger effect size than document interventions.

#### Dependent Measures

Participants' behavior change was assessed using subjective self-report measures, such as self-reported physical activity (Marquardt et al., 2017) or unhealthy snacking (Adriaanse et al., 2010), and objective measurement, such as attendance to a class session (Sailer et al., 2015) or course completion (Kizilcec and Cohen, 2017).

#### Expectations of Success

Expectations of success, perceived likelihood of attaining the desired outcome (i.e., general expectations, usually measured by “how likely do you think it is that you will attain your goal?”) moderate the effect of mental contrasting on goal pursuit (Oettingen, 2000; Oettingen et al., 2001). When expectations of success are high, people will firmly commit to pursuing the desired outcome; when expectations of success are low, people will postpone or abandon the achievement of their goals (Oettingen, 2012). The effect of MCII on goal commitment was also hypothesized to be moderated by expectations, that is, higher expectations enhance the effect of MCII.

## Method

### Literature Search and Criteria for Inclusion

To locate relevant studies, we searched English (SCOPUS, PsyINFO, PsyARTICLE, PubMed, Web of Science, Science Direct, Springer Link, ProQuest Dissertations & Theses, and Google Scholar) and Chinese electronic databases (China National Knowledge Infrastructure, Wanfang Database, and VIP Paper Check System) using the search terms “mental contrasting,” “implementation intention,” “WOOPs (short for wish-outcome-obstacle-plan),” “if–then plan,” and “plan.”

**Figure 1:**
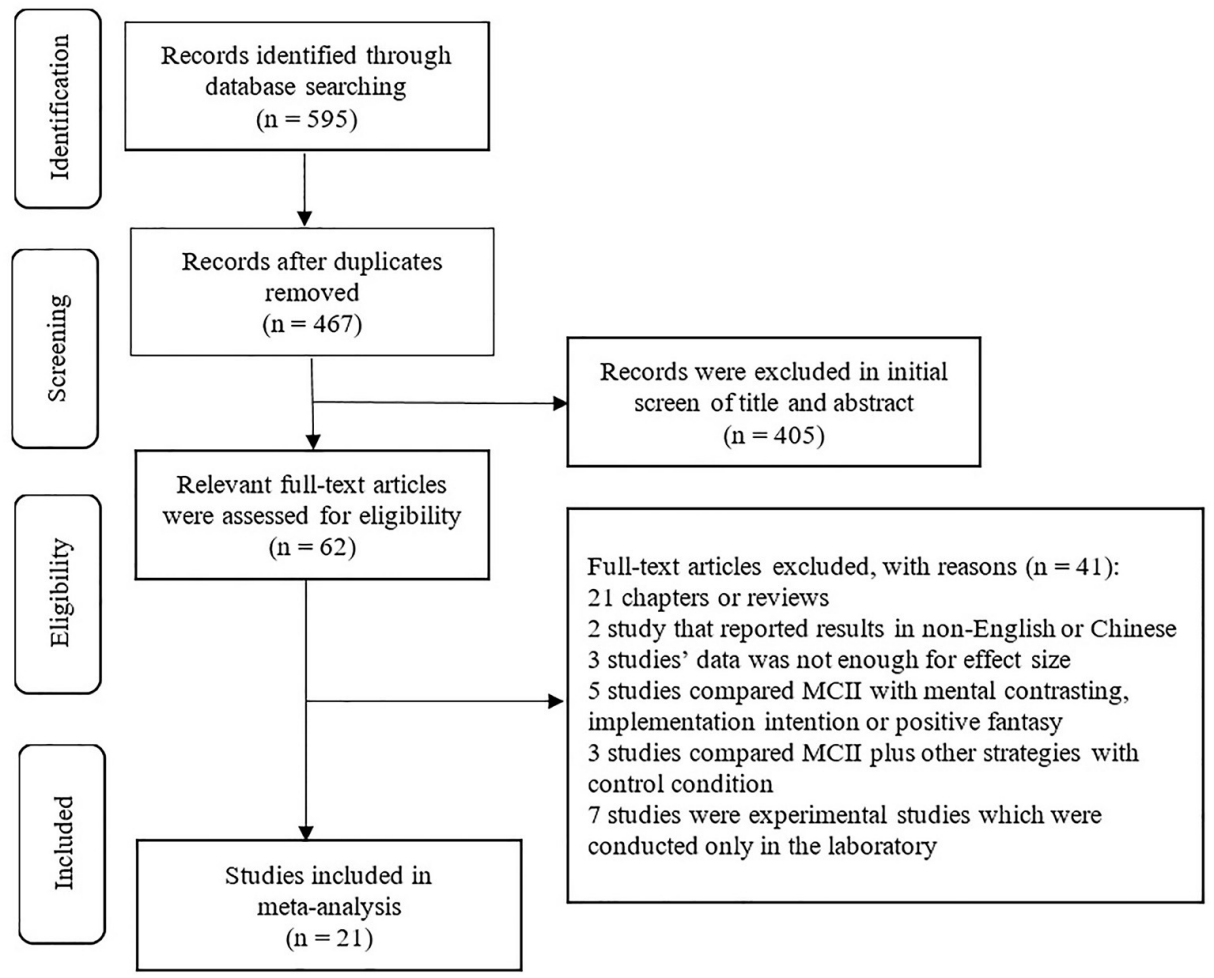
Flow diagram of the literature search and study selection process.

Criteria for inclusion were as follows: (1) the study was empirical—review studies were excluded; (2) the available data were sufficient for calculating an effect size; (3) the study reported methods and results in English or Chinese; (4) the study was field intervention research—experiments conducted only in laboratories were excluded [unlike the meta-analysis by Gollwitzer and Sheeran (2006), which included field intervention studies, related studies, and laboratory studies]; and (5) the intervention was delivered in the context of an intervention and a control condition, and the difference between groups was whether MCII was implemented or not. The included studies were divided into two groups, based on the differences in condition manipulation. In the first group, studies compared MCII intervention conditions with no treatment control conditions (e.g., Houssais et al., 2013; Gollwitzer et al., 2018). Studies in the second group compared MCII plus additional behavior change technique conditions with additional behavior change technique conditions, containing related information (e.g., Stadler et al., 2009; Sailer et al., 2015; Marquardt et al., 2017), theory of planned behavior (Hawes, 2007), and self-regulation assessment (Gawrilow et al., 2013). Finally, 24 independent studies from 21 articles were included. Figure 1 presents a flow diagram for the literature search showing the number of studies identified, screened, found to be eligible, and finally included in the meta-analysis.

### Literature Coding

The following variables from 24 independent studies were coded for the meta-analysis procedure: study information (authors, published year), sample size, sample age (children, college students, adults from general population, physically or psychologically ill adults), publication status (published article, unpublished master's thesis, and doctoral dissertation), goal domain (health, academic, relationship, personal), type of intervention (experimenter intervention, document intervention), dependent measures (self-report, objective), and goal success expectation.

### Data Analysis

For the effect size from each study, we used the standardized mean difference (Hedges' *g*) to estimate the training effect between the MCII intervention group and the control group. Several studies reported multiple outcome variables or used a tracking measurement design; however, using multiple effect sizes from the same independent sample would violate the independence assumption, lead to excessive performance of individual studies, and result in a deviation of the results. Therefore, it was necessary to combine multiple outcomes reported in one study so that only one effect size was obtained from each independent sample (Lipsey and Wilson, 2001; Ellis, 2010). We averaged the effect sizes of multiple outcomes in one study. For the list and details of the studies included in the meta-analysis, see Table 1.

**Table 1:** Studies included in the meta-analysis: effect size and study characteristics.

**Studies**	**Sample size**	**Sample ages**	**Goal domain**	**Type of intervention**	**Expectation level**	**Dependent measure**	**Effect size (Hedges' *g*)**	**95% CI**
Abbott et al. (2020)	27	General adult	Health	Experimenter intervention	Not available	Objective+Self-report	0.44	(−0.35, 1.22)
Adriaanse et al. (2010) study 1	51	College students	Health	Experimenter intervention	Not available	Self-report	0.48	(−0.08, 1.04)
Christiansen et al. (2010)	60	Adult patient	Health	Experimenter intervention	Not available	Objective+Self-report	0.27	(−0.24, 0.78)
Duckworth et al. (2011)	66	Children	Academic	Document intervention	4.66	Objective	0.52	(0.03, 1.01)
Fritzsche et al. (2016)	47	Adult patient	Health	Experimenter intervention	6.25	Self-report	1.14	(0.41, 1.87)
Gawrilow et al. (2013)	116	Children	Academic	Experimenter intervention	Not available	Objective	0.16	(−0.20, 0.52)
Gollwitzer et al. (2018)	68	General adult	Health	Document intervention	Not available	Self-report	0.51	(0.05, 0.96)
Hawes (2007)	124	16–62 years	Health	Document intervention	Not available	Objective	−0.03	(−0.51, 0.44)
Houssais et al. (2013)	80	College students	Relationship	Experimenter intervention	4.08	Self-report	0.61	(0.16, 1.06)
Kizilcec and Cohen (2017) study 1	9619	Not available	Academic	Document intervention	Not available	Objective	0.09	(−0.01, 0.19)
Kizilcec and Cohen (2017) study 2	4290	Not available	Academic	Document intervention	Not available	Objective	0.06	(−0.02, 0.13)
Loy et al. (2016)	55	College students	Health	Experimenter intervention	Not available	Self-report	0.54	(0.00, 1.08)
Marquardt et al. (2017)	183	Adult patient	Health	Experimenter intervention	Not available	Self-report	0.47	(0.06, 0.88)
Oettingen et al. (2015) study 1	56	College students	Personal	Experimenter intervention	4.92	Self-report	0.60	(0.06, 1.13)
Oettingen et al. (2015) study 2	40	College students	Personal	Experimenter intervention	Not available	Self-report	0.97	(0.31, 1.63)
Saddawi-Konefka et al. (2017)	34	College students	Academic	Experimenter intervention	Not available	Self-report	0.67	(0.03, 1.32)
Sailer et al. (2015)	36	Adult patient	Health	Experimenter intervention	Not available	Objective	0.56	(−0.04, 1.15)
Scheurnschloß (2017) study 3.3	163	General adult	Personal	Document intervention	5.87	Self-report	0.30	(−0.01, 0.60)
Stadler et al. (2009)	256	General adult	Health	Experimenter intervention	Not available	Self-report	0.48	(0.23, 0.72)
Velasquez-Sheehy (2015)	117	Children	Academic	Experimenter intervention	4.35	Objective	0.32	(−0.04, 0.69)
Wang and Gai (2016)	81	College students	Academic	Document intervention	5.58	Self-report	−0.11	(−0.54, 0.33)
Wang (2014) study 4	91	College students	Personal	Experimenter intervention	5.54	Self-report	0.46	(0.02, 0.90)
Wang (2014) study 5	47	College students	Health	Experimenter intervention	5.54	Self-report	0.17	(−0.47, 0.81)
Wittleder et al. (2019)	200	Generaladult	Health	Document intervention	Not available	Self-report	0.31	(0.14, 0.47)

*Adult patient indicates adults with physical or psychological illnesses, and general adults indicates adults from general population. Expectation level indicates levels of expectations of success*.

To calculate the results of the meta-analysis, we could have chosen a fixed-effects model or random-effects model, which is dependent on whether the same true effect size was expected for the different studies. The fixed-effects model assumes that different studies have the same true effect size, while the random-effects model assumes that different studies have different true effect sizes (Borenstein et al., 2009). Because we hypothesized that there were different effect sizes based on the different literature coding categories, we used random-effects models.

The rationality of model selection was verified through a heterogeneity test using *Q* and *I*^2^ indexes. *Q* was used to determine whether the heterogeneity of the effect size distribution was significant, and *I*^2^ described the percentage of total variation across studies due to heterogeneity rather than chance; 25, 50, and 75% were the boundaries for a low, medium, and high degree of heterogeneity, respectively (Higgins et al., 2003).

### Publication Bias Analysis

Publication bias threatens the validity of published research by masking small and null effects; therefore, publication bias was evaluated. This study used four types of publication bias analyses: funnel plot, Rosenthal's fail-safe N, Egger linear regression test, and trim-and-fill method.

Comprehensive Meta-Analysis 2.0 (CMA 2.0) was used to analyze the data.

## Results

### Main Effect

The model included 24 independent studies from 21 articles (*N* = 15,907). Due to the significant medium level of heterogeneity [*Q* (23) = 56.540, *I*^2^ = 59.321%, *p* < 0.001], we used the random-effects model. Overall, MCII yielded a small to medium significant effect size, *g* = 0.336, 95% CI (0.229, 0.443).

### Publication Bias Analysis

**Figure 2:**
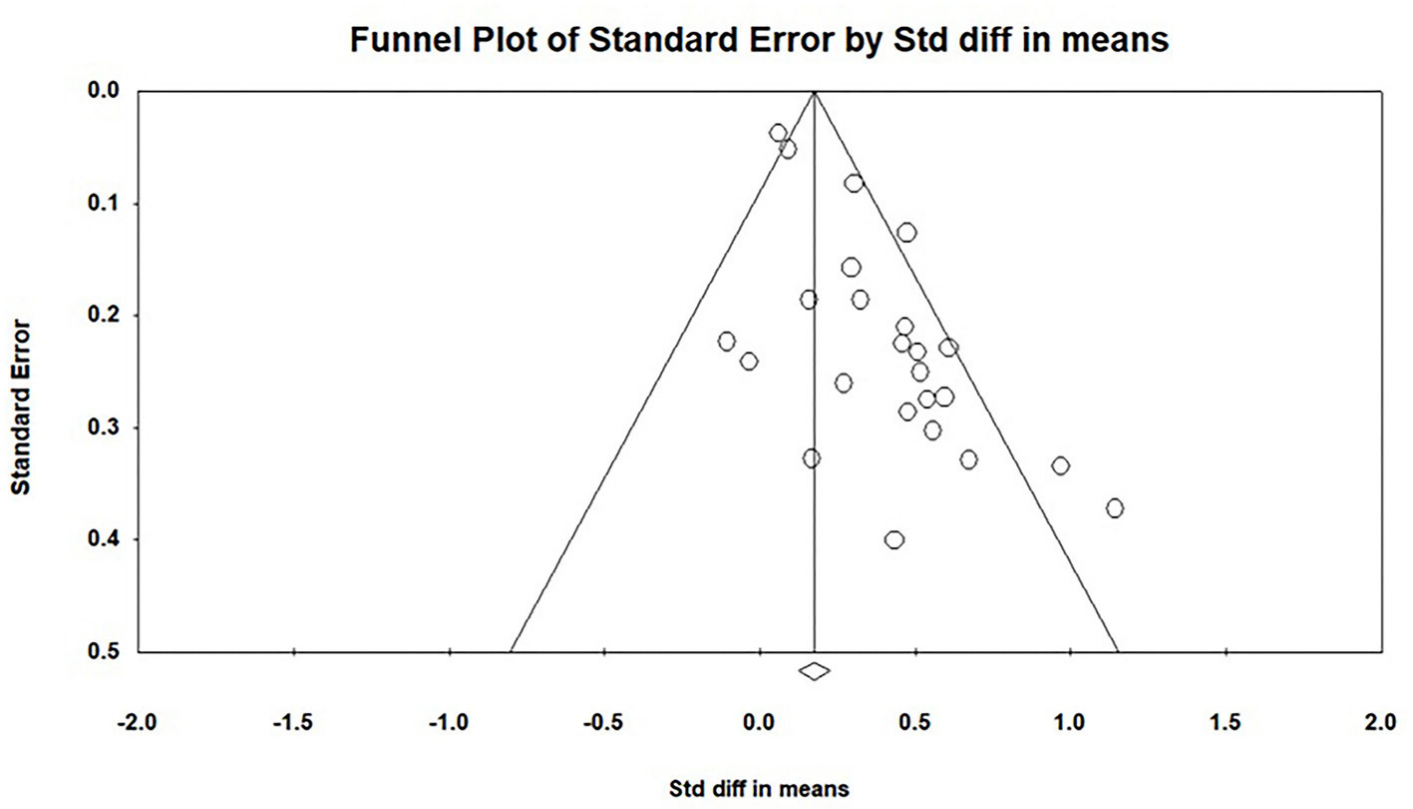
Funnel plot of the MCII strategy on goal attainment.

The funnel plot of the effect of MCII on goal attainment showed strong asymmetry (see Figure 2), with more studies on the right side than on the left, indicating that some studies with nonsignificant results or small effect sizes may have been missing from the left side, which could indicate publication bias. Fail-safe N for this study was 482, greater than the critical value of 130 (5*k* + 10), indicating that the results were reliable, and there was no publication bias. Egger's regression test (*t* = 5.46, *p* < 0.01) indicated that there might be publication bias. Finally, we used the “trim-and-fill” method to trim eight studies, and the adjusted values were 0.242, 95% CI (0.143, 0.342). Five unpublished master's theses and doctoral dissertations were included in the meta-analysis, accounting for 21% of the corpus—sufficient to eliminate publication bias. In conclusion, while analyses indicated that the current study might have had some publication bias, the main conclusion of this meta-analysis was found to be relatively effective and reliable.

### Moderator Analysis

The moderator analysis examined the effect of MCII across two publication statuses, four sample ages, four goal domains, two intervention types, two dependent measures, and two expectation levels. For the moderator analysis, we decided to exclude two independent studies with a large sample size (13,909 participants in total, accounting for 87.44% of the whole sample in the current meta-analysis) by Kizilcec and Cohen (2017), comprising 8 min online document interventions with objective-dependent measures (MOOCs completion rate), conducted in the academic context. As the sample size in this article would account for more than 90% of the sample size in the respective subgroup when performing the moderator analysis, the effect size for online document interventions, object-dependent measures, and academic context would be mainly derived from a single article. As moderator variables covary across studies (Lipsey, 2003), these studies may cause the results of any analysis focusing on a single moderator variable to be misleading.

The results (Table 2) showed no significant differences in effect size between published papers and unpublished master's theses and doctoral dissertations, further indicating a low possibility of publication bias.

**Table 2:** Moderators of effect size.

**Moderator**	***k***	***N***	***g***	**95% CI**	**Heterogeneity**		
					***Q***	***df***	***p***
***Publication status***					1.587	1	0.208
Published	17	1,456	0.404	(0.309, 0.498)	18.780	16	0.280
Unpublished	5	542	0.271	(0.089, 0.454)	2.486	4	0.647
***Sample ages***					2.201	3	0.532
General adult	5	714	0.359	(0.240, 0.478)	1.866	4	0.760
Children	3	299	0.301	(0.073, 0.530)	1.332	2	0.514
Adult patient	4	326	0.521	(0.258, 0.785)	3.790	3	0.285
College student	9	535	0.447	(0.272, 0.622)	10.705	8	0.269
***Goal domain***					3.075	3	0.380
Academic	5	414	0.255	(0.062, 0.449)	5.731	4	0.220
Health	12	1,154	0.379	(0.273, 0.485)	10.389	11	0.496
Personal	4	350	0.457	(0.241, 0.673)	3.658	3	0.301
Relationship	1	80	0.609	(0.160, 1.058)	0	0	1.000
***Type of intervention***					4.797	1	0.029
Document intervention	6	702	0.277	(0.154, 0.399)	6.617	5	0.251
Experimenter intervention	16	1,296	0.465	(0.349, 0.580)	11.439	15	0.721
***Dependent measure***					1.403	1	0.236
Objective	5	459	0.272	(0.078, 0.467)	3.878	4	0.423
Self-report	15	1,452	0.403	(0.308, 0.499)	17.386	14	0.236
***Expectation level***					1.466	1	0.226
High	5	429	0.297	(0.097, 0.496)	9.099	4	0.059
Low	4	319	0.481	(0.258, 0.705)	1.221	3	0.748

*k = number of studies, N = sample size, Q represents Q heterogeneity test. Publication status: published means published articles; unpublished means unpublished thesis and dissertation. In the sample ages, three studies were excluded because one study consisted of both children and adults, and two studies did not report the age of the sample. In dependent measures, 1 study was excluded because this study consisted of both self-report and objective measures. At the expectation level, we used expectations of success (measured by “how likely do you think it is you will…,” Likert 7-point scales) as indicators and defined 5 and above as high expectation and lower than 5 as low expectation. Thirteen studies were excluded because they did not report expectations of success. Two independent studies by Kizilcec and Cohen (2017) were excluded from moderator analysis*.

The moderator analysis found no significant moderating effect of sample age on MCII, indicating that the strategy was effective for individuals of different ages. Although the effect size in the academic context (*g* = 0.255) was relatively lower than in other contexts, the difference was not significant [*Q* (3) = 3.075, *p* = 0.380].

Type of intervention was a significant moderator, and document interventions (*g* = 0.277) had a significantly lower effect size than experimenter interventions [*g* = 0.465, *Q* (1) = 4.797, *p* < 0.05].

For the dependent measures, there was no significant difference between the effect of MCII interventions on self-reported results and the effect of MCII interventions on objective results. The expectation level was not a significant moderator either, and the effect size of MCII on high expectation (*g* = 0.297) and low expectation goals (*g* = 0.481) did not differ significantly.

## Discussion

### Intervention Effect of MCII on Goal Attainment

This research evaluated the effect of MCII on goal attainment, using 24 independent studies from 21 articles, with a total effect size of small to medium, *g* = 0.336. This is similar to Cross and Sheffield's (2019) meta-analysis, which found that MCII had a small-to-medium effect on health behavior change. As expected, although several studies found that MCII was more effective than mental contrasting or implementation intention alone (Adriaanse et al., 2010; Kirk et al., 2013), and mental contrasting alone or implementation intentions alone intervention studies found small-to-medium effect size (Belanger-Gravel et al., 2013; Carrero et al., 2019; Cross and Sheffield, 2019), MCII interventions had a small-to-medium effect size on behavior change.The first possible reason for this finding is that the effects of mental contrasting or implementation intentions are moderated by other variables; for example, mental contrasting can encourage individuals with low expectations of success to let go their wishes (Oettingen and Cachia, 2016), and implementation intentions has no promoting effect on goals for highly impulsive individuals (Churchill and Jessop, 2010). A combination of these two strategies can be moderated by even more variables, which may decrease the effect size. Second, studies that found MCII to be more effective than its component processes (Adriaanse et al., 2010; Kirk et al., 2013) compared MCII with mental contrasting or implementation intention in the same study, while the meta-analysis research on MCII or mental contrasting/implementation intentions were based on different studies. Therefore, more studies that compare MCII with its component processes in the same study are needed to make conclusions.

This meta-analysis found MCII to be a concise, low-cost, and effective self-regulation strategy for goal achievement, which can be used to promote goal attainment. Although we found that effect size of MCII interventions was similar to interventions employing either mental contrasting or implementation intentions, we recommend using MCII as mental contrasting and implementation intention complement each other (Kappes et al., 2012b, 2013; Oettingen, 2012).

### Moderators of the Effect of MCII on Goal Attainment

The current meta-analysis examined six potential moderating variables: publication status, sample age, goal domain, type of intervention, dependent measure, and goal success expectation. Of these, only intervention type was found to be a significant moderator.

MCII interventions based on experimenter interventions had a significantly higher effect size than document interventions. On the one hand, face-to-face interventions could promote effectiveness by increasing interpersonal relationship (Elder et al., 2005). On the other hand, participants may formulate low-quality MCII strategies in a free document-writing environment (Kizilcec and Cohen, 2017) and may have low commitment to the MCII strategy, which will reduce its effectiveness (de Nooijer et al., 2006). Furthermore, while face-to-face interventions were more effective, cost-saving document-based interventions (such as online interventions) had a small to medium effect size on behavior change (*g* = 0.277) and are feasible when face-to-face interventions are difficult to implement.

We did not find a significant moderating effect for publication status, indicating that the effect of MCII was not exaggerated in published articles. In addition, the effect size did not vary with sample age, goal domain, or dependent measures, revealing that MCII was effective for people of different ages, for goal pursuit in different domains, and for different measures of behavior change. However, as the number of studies in the current meta-analysis was not large, more studies are needed to draw conclusions.

We did not find a moderating effect for expectation level in this study, due to the ceiling effect and low variance of expectation. On the one hand, most intervention studies in this meta-analysis were designed to promote goal attainment and explicitly required the participants to choose feasible goals for themselves (Stadler et al., 2009, 2010; Adriaanse et al., 2010; Duckworth et al., 2013; Gawrilow et al., 2013; Houssais et al., 2013; Sailer et al., 2015; Fritzsche et al., 2016), and participants' expectations of success in the existing studies were all above 4 points (7 points), which was above the average level. Therefore, the studies included in this meta-analysis guaranteed that the participants had relatively high success expectations in each study. On the other hand, only nine studies reported expectations of success in their study, and the variance in expectation was small, so more studies are needed to find the moderator role of expectation.

The results of moderator analysis should be interpreted with caution due to the small number of studies included in the meta-analysis and covarying moderator variables across studies (Lipsey, 2003), which may cause misleading results in analyses focusing on single moderator variables.

### Future Research Direction

Some potential influencing factors of the effect of MCII on goal achievement were not examined in this study. First, the duration of the intervention should be considered. The long-term effects of MCII may be better than the short-term effects, and several longitudinal health studies found that MCII was more conducive to the long-term retention of behavioral change compared to the control group and helped individuals respond flexibly to changes in circumstances (Stadler et al., 2009, 2010). Due to the small variation in duration in the current follow-up studies, this meta-analysis failed to determine the difference in effect sizes at different time points, which can be tested in future studies. Second, the frequency of MCII strategy usage was left unexplored. MCII can help individuals foster a habit of moving toward a goal, and the frequency of using the strategy will affect the strength of habit. A meta-analysis by Da Silva et al. (2018) found that an implementation intention intervention without reinforcement had no significant effect on adult physical activity, while an implementation intention intervention with reinforcement had a small to medium effect size on adult physical activity. Most previous studies allowed subjects to complete the application of MCII strategy in the intervention stage only, and only a few encouraged participants to practice MCII strategy every day (Gollwitzer et al., 2018). Upon the completion of future studies, the moderating effect of the frequency of MCII use on the intervention effect can be tested. Third, cultural differences in the effects of MCII strategies should be considered (Oettingen, 1997; Oettingen et al., 2008). Most MCII intervention studies focused on the Western culture. Kizilcec and Cohen's (2017) studies on completing MOOCs included Western and Eastern subjects and found MCII to be effective for learners in individualist cultures but not learners in collectivist cultures. The completion of MOOCs is an individual goal; in collectivist cultures, learners' obstacles might conflict with their cultural norms (i.e., participants may have to be there for the group but not for their personal needs), which could render the obstacles insurmountable in collectivist cultures. MCII should not lead to enhanced effort when members of collectivist cultures focus on individualist wishes, but it should increase effort and goal attainment when people focus on their collectivist wishes. We assumed that MCII could have the similar effect size on collectivist wishes and goals in collectivist cultures as on individualist wishes and goals in individualist cultures. Future studies should further explore MCII in Eastern cultures, with researchers guiding participants to match their wishes and obstacles (i.e., matching individual wishes with individual obstacles, and collectivist wishes with collectivist obstacles) during the intervention procedure.

Although the overall effect size of the MCII strategy was small to medium, as expected, future studies need to optimize MCII intervention effect in three ways. First, experimenters should participate in the intervention procedure and conduct face-to-face interventions with participants to ensure the formation of high-quality MCII strategies. Second, to prevent participants from forgetting their MCII strategies, future studies could increase the frequency of their application. For example, experimenters can conduct multiple interventions or remind participants to use the MCII strategy multiple times. The optimal interval between each MCII intervention or reminder also needs to be explored in future studies. Third, the flexibility of MCII strategy should be improved. A concrete plan would promote recognition of the planned cues but would make it more difficult to notice other available cues, and strictly rigid self-regulation strategies may hinder goal attainment (Parks-Stamm et al., 2007). Thus, flexibility of the MCII strategy is essential; mental contrasting can lead individuals with high expectations to flexibly detect potential obstacles in the context (Kappes et al., 2013), which is the premise for developing flexible implementation intention, and experimenters can facilitate the formation of implementation intentions to improve the MCII strategy (i.e., “if one MCII strategy fails, I'll form a new MCII strategy”).

### Limitations

This study had a few limitations. The number of studies involved in this meta-analysis was small. This might be a reason why we did not find a significant moderator effect for sample age, goal domains, dependent measures, or expectation levels. Moreover, in the moderator analysis, we excluded Kizilcec and Cohen (2017) because of the large sample size in their study, which might render the results of moderator effect unreliable. More MCII intervention studies from different research groups, in a variety of settings, and with different participant groups, especially studies with sample sizes proportionate to that of Kizilcec and Cohen's (2017) study, are needed to strengthen the efficacy of moderator variables.

## Conclusions

In this study, the effects of MCII on goal achievement, along with its moderating factors, were evaluated through meta-analysis. The following conclusions were reached: (1) MCII can effectively promote individual goal achievement, with a small to medium overall effect size (*g* = 0.336), and (2) the effect of MCII may be moderated by intervention type, and more studies are needed to strengthen the case for the efficacy of moderator variables.

## Data Availability Statement

The raw data supporting the conclusions of this article will be made available by the authors, without undue reservation.

## Author Contributions

GW analyzed the data, drafted the manuscript, and revised the manuscript several times. YW did the literature search and study selection. XG reviewed the article and gave many useful suggestion to make the manuscript much better. All authors contributed to the article and approved the submitted version.

## Conflict of Interest

The authors declare that the research was conducted in the absence of any commercial or financial relationships that could be construed as a potential conflict of interest.
